# Improving Compliance With Operative Note Guidelines Through the Implementation of an Electronic Proforma

**DOI:** 10.7759/cureus.32222

**Published:** 2022-12-05

**Authors:** Emmanuel O Oladeji, Smriti Singh, Konstantinos Kastos

**Affiliations:** 1 General Surgery, Epsom and St Helier Hospital, Carshalton, GBR

**Keywords:** operative note, surgical trainee, general surgery, patient safety, guideline

## Abstract

Background

An operative note (op note) is a vital medical record of remarkable clinical, medico-legal and academic relevance. The Royal College of Surgeons (RCS) has set out a guideline to standardise op notes. This closed-loop audit assessed the compliance of op notes completed in our local hospital against the guidance set by RCS with the view to identify deficiencies and improve practice.

Methods

A retrospective review of general surgery operative notes was carried out to access their various characteristics against RCS guidance. Two additional parameters were assessed, namely, ‘cadre of the surgeon that completed the op note’ and ‘use of abbreviations’. To improve compliance with RCS guidelines, an electronic proforma (EP) that included all the 18 characteristics listed in good surgical practice was implemented and a re-audit was undertaken six months afterwards.

Results

A total of 200 op notes were reviewed, 98 during the initial audit cycle and 102 at the re-audit. Seventy-eight per cent (78%) of the op notes were written by trainees. At the initial audit, seven parameters performed poorly, with compliance ranging between 5.1% and 76.5%. The re-audit demonstrated improved adherence to guidelines following the implementation of the EP, as well as a reduction in the use of abbreviations. The overall compliance improved from roughly 80% to >95%.

Conclusion

A sustainable change was achieved through the implementation of EP with improvement demonstrated in content and structure. The need to provide teaching to trainees who are responsible for writing a vast majority of op notes was identified.

## Introduction

Operative notes (op notes) are vital medical records, which serve as a fundamental means of communicating operative findings and procedure details and provide information on post-operative patient care. The safe onward care of surgical patients depends significantly on the accuracy and legibility of operation notes. When not properly done, an op note can be a source of miscommunication and mistakes, can jeopardise patient postoperative management and overall patient experience and can contribute to patient morbidity and mortality. It is also an invaluable legal document and may be the only tenable defence for the surgeon, should a medico-legal enquiry arise [1]. Furthermore, failure to make accurate documentation of procedures could lead to inappropriate remuneration for procedures undertaken in a hospital [2]. It goes without saying that, given the clinical, academic and medico-legal ramifications of op notes, maintaining a full and proper record of an operation is the professional responsibility of every surgeon [1].

In a bid to standardize the content of operation notes, the Royal College of Surgeons (RCS) published a guideline embedded in the document named ‘good surgical practice’ in 2014 [3]. The guideline is instructive on documentation of sufficient details to enable continuity of care, using an 18-item template This was followed by several audits assessing the compliance of different centres with this guideline [4,5]. The imperative to improve the quality of op notes had also been alluded to by the National Confidential Enquiry into Peri-Operative Deaths (NCEPOD) with a mandate to address the sub-standard state and significant variation in the quality of op notes across different hospitals [6]. We conducted a closed-loop audit to assess the quality of general surgical operative notes completed in our local hospital against the guidelines set by RCS [3], with the view to identify deficiencies, educate clinicians regarding this guidance and improve practice.

## Materials and methods

We carried out a closed-loop retrospective audit on general surgery operative notes completed at our local hospital. A mix of 100 upper gastrointestinal and colorectal procedures performed between November 2020 and December 2020 was included in the initial audit. Two op notes were not available, hence 98 were assessed. The 98 op notes were audited against the 18 characteristics set by RCS (Table 1).

**Table 1:** RCS guideline for operative notes as outlined in good surgical practice *RCS - Royal College of Surgeons*; *DVT - deep venous thrombosis*

RCS guideline for operative notes
1. Date and time
2. Elective/emergency procedure
3. Names of operating surgeon and assistant
4. Name of theatre anaesthetist
5. Operative procedure carried out
6. Incision
7. Operative diagnosis
8. Operative findings
9. Any problems/complications
10. Any extra procedure and the reason why it was performed
11. Details of tissue removed, added or altered
12. Identification of any prosthesis used, including the serial numbers of the prosthesis and implanted materials
13. Details of closure technique
14. Anticipated blood loss
15. Antibiotic prophylaxis
16. DVT prophylaxis
17. Detailed postoperative instructions
18. Signature

Two additional parameters were assessed, namely, ‘cadre of the surgeon that completed the op note’ and ‘use of abbreviations’. Operative notes were retrieved from the electronic medical record software and evaluated by four junior doctors in the department of general surgery. Hundred per cent (100%) was the expected standard. The results of the initial audit were presented at the hospital clinical governance meeting, which was well-attended by surgeons at all levels. The results were discussed in detail and strategies to improve compliance with the guidance set by ‘best surgical practice’ were highlighted. An action plan was agreed upon, which included wider dissemination of audit results to all cadres of the surgeon, education on doing notes electronically and introduction of aide-mémoires in theatres and surgeons’ offices. The most pivotal strategy was to introduce an electronic proforma (EP) that had all the 18 elements listed in good surgical practice, thereby ensuring none of the details gets missed. It was decided to re-audit the practice six months after the implementation of an intervention. The EP template was developed in collaboration with the information technology (IT) department and implemented for use. A re-audit was conducted six months later with 105 op notes included. Three were unavailable, leaving 102 to be analysed. Data were extracted and analysed using Microsoft Excel software (Microsoft Corporation, Redmond, WA). The findings of the closed-loop audit were presented at the departmental quality control meeting and further learning points were discussed.

## Results

**Figure 1:**
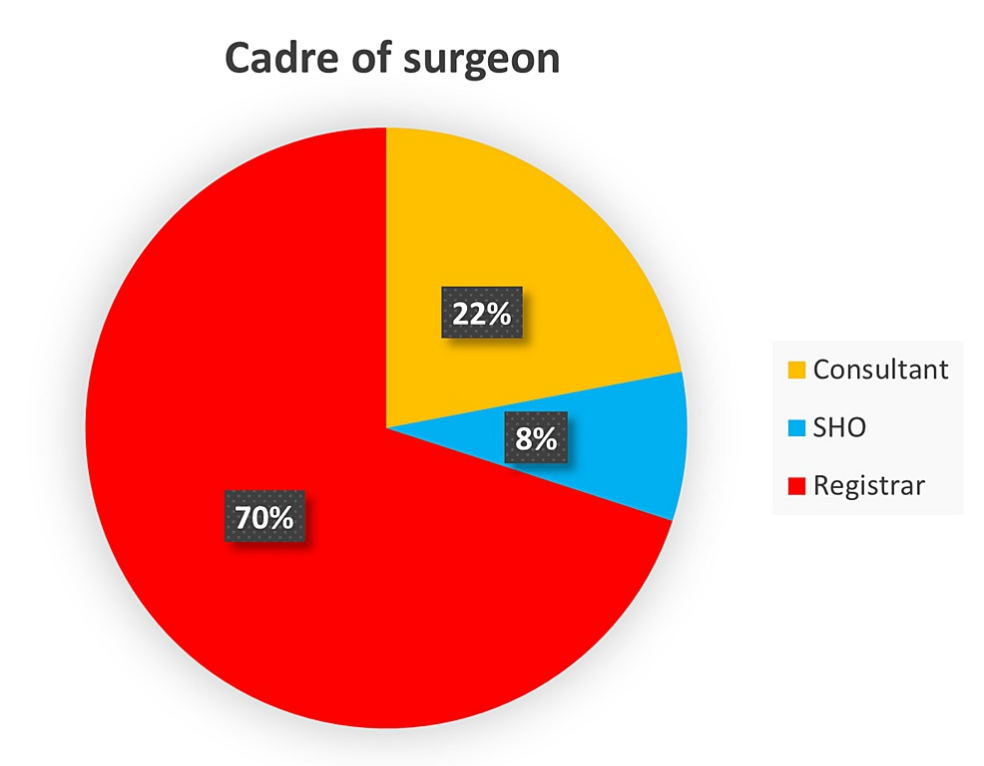
Proportion of op notes written by surgeon cadre SHO - senior house officer

A total of 200 op notes on upper gastrointestinal and colorectal procedures were reviewed, 98 during the initial audit cycle and 102 at the re-audit. Seventy per cent (70%) of the op notes were completed by a registrar (middle-grade doctor), 22% by a consultant and 8% by a senior house officer (SHO; junior doctor) (Figure 1).

Hundred per cent (100%) adherence to the RCS guideline was considered the standard. From the initial audit cycle, 11 of the 18 parameters performed excellently, with 100% adherence to the documentation of ‘date and time’, ‘whether elective/emergency procedure’, ‘names of operating surgeon’, ‘operative procedure carried out’, ‘incision’, ‘operative findings’, ‘any problems/complications’, ‘any extra procedure and the reason why it was performed’, ‘details of closure technique’, ‘postoperative instructions’ and ‘signature’. Seven neglected parameters were identified, including anaesthetist name (67.3%), operative diagnosis (78.6%), tissue removed or added (52%), prosthesis (52%), estimated blood loss (5.1%), antibiotic prophylaxis (52%) and deep venous thrombosis (DVT) prophylaxis (31.6%) (Table 2).

**Table 2:** Summary of parameters’ performance at the first and second audit cycles

Parameter	Frequency (%)	
	Initial Audit	Re-audit
Date and time	100	100
Elective/emergency	100	100
Names of operating surgeon	100	100
Name of theatre anaesthetist	67	56.9
Operative procedure carried out	100	100
Incision	100	100
Operative findings	100	100
Operative diagnosis	76.5	65.7
Any problems/complications	100	100
Any extra procedure performed with reason	100	100
Details of tissue removed, added or altered	63.75	97.5
Identification of any prosthesis or implanted material used	63.75	96
Details of closure technique	100	100
Anticipated blood loss	5.1	98.7
Antibiotic prophylaxis	63.73	98.7
DVT prophylaxis	31.6	100
Postoperative instructions	100	100
Signature	100	100

**Figure 2:**
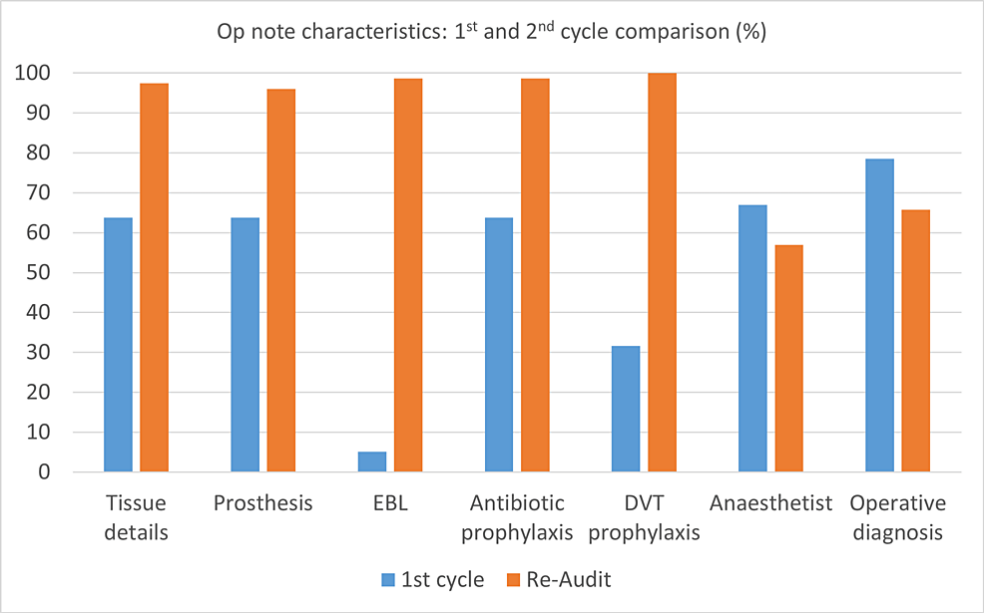
Comparison of op note characteristics; pre and post-intervention EBL - estimated blood loss, DVT - deep venous thrombosis

The second audit cycle demonstrated a significant improvement in the level of compliance of most of the parameters that scored poorly in the first audit cycle. The details of ‘tissue removed, added or altered’ was documented in 97% (52) of op notes, ‘identification of any prosthesis used, including the serial numbers’ in 96% (52), ‘estimated blood loss’ in 98.7% (5.1), ‘antibiotic prophylaxis’ in 98.7% (52) and ‘DVT prophylaxis’ in 100% (31.6) (Figure 2). There was a worsening of documentation of the anaesthetist name to 56.9% (67.3), operative diagnosis to 65.7% (76.5) and the operating assistant was missing from 25% of op notes. The 11 parameters that performed satisfactorily at the initial audit retained this fine quality. The overall level of compliance improved from about 80% at the initial audit to >95% at the re-audit.

## Discussion

**Figure 3:**
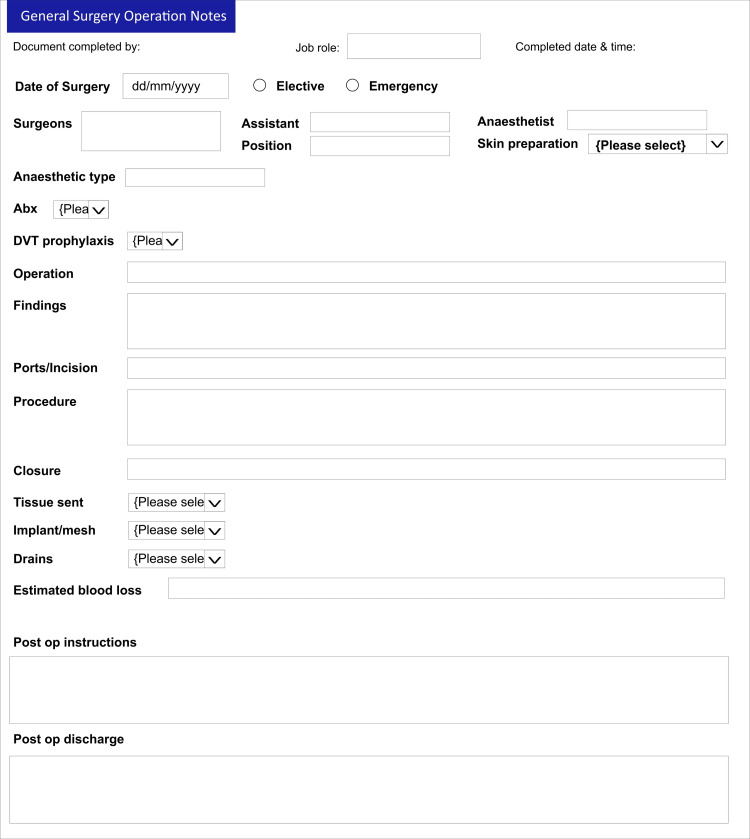
The new improved operative note proforma DVT - deep venous thrombosis, Abx - antibiotics

**Figure 4:**
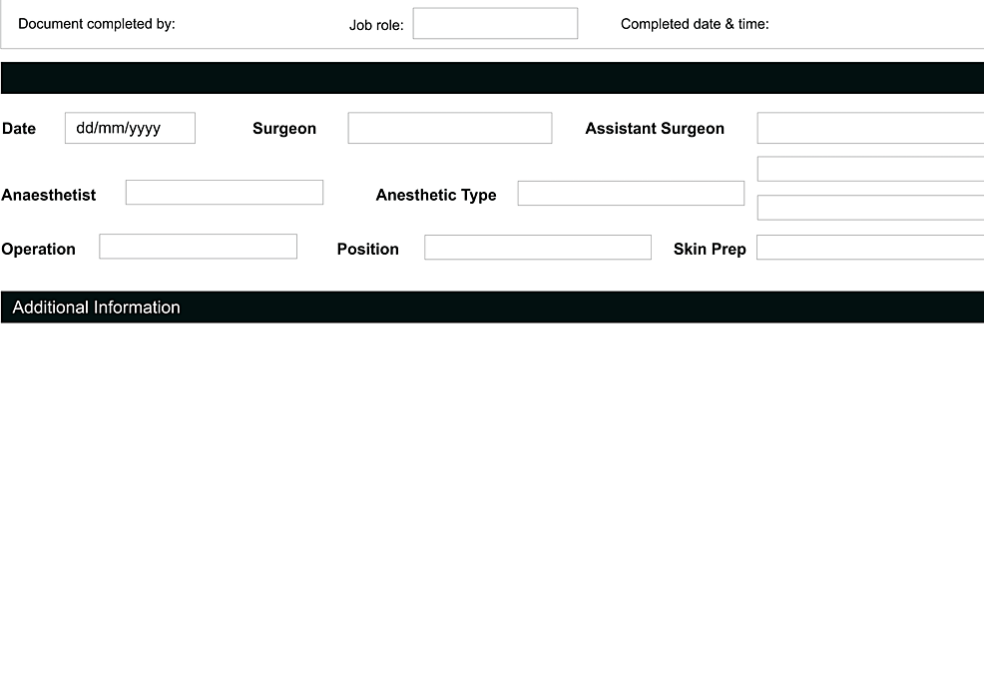
Operative note template in use prior to the initial audit

Operative notes are an essential document for the post-operative management of patients [7]. The RCS guideline is instructive about the prescription for legibility, details of information and timing of completion of op notes [3,8]. As evident from our result, 78% of the op notes were written by the registrar and junior trainees (Figure 1) who may not have received any formal training [7,9]. The vital need to incorporate learning on the ethical approach to the writing of operation notes into core surgical skills teaching for trainees has been highlighted in previous studies, and this was emphasized at our clinical governance meeting [10,11]. Making sure that senior surgeons review the op notes written by the junior trainees is also a useful practice worthy of consideration. From this closed-looped audit, there was overall increased compliance and documentation was noted to be more accurate. The biggest improvement was seen in the documentation of estimated blood loss, recorded in 98.7% at the re-audit as against 5.1% during the first audit cycle. The adherence of four other categories significantly improved to >96% for tissue removed, added or altered; details of prosthesis used; antibiotic prophylaxis; and 100% for DVT prophylaxis. This improvement is most likely due to better awareness and implementation of our upgraded system, which ensures all operative notes are completed electronically using the new operative note template, which has all the important characteristics (Figure 3) as against the one in use prior to the initial audit, which had no such structure (Figure 4).

Previous work has shown improvements in the quality of operative notes by using a surgical electronic template [12-14], and this practice should be advocated to improve standards and ensure patients are properly looked after. There was less compliance seen for documenting the name of the anaesthetist while the operating assistant was not stated in 25% of op notes. One-third of these were major procedures that could not have been undertaken by one surgeon. Understandably, some minor procedures could potentially be carried out without an assistant, and not all sections are applicable in all procedures, but in the absence of documentation to provide clarity, the relevance or otherwise of the missing details will only be a subject of conjecture. To address this, we recommended that surgeons should state ‘not applicable’ in sections that are not relevant to the procedure, including assistant and closure (if only dressings were applied, this should be clearly stated). Worsening of documentation of the aforementioned parameters was highlighted and surgeons were reminded that every section of the operation note holds a vital bearing for patient care. Failure to document the closure technique, for instance, may lead to patients being mistakenly advised to return to their general practitioner (GP) for suture removal. This would cost the patient an avoidable trip to the hospital, distress from finding out the obvious, unnecessarily add to the overwhelming pressure experienced by GPs and an additional patient carbon footprint. One way we proposed to avoid missing details in op notes was to ensure that all sections in the operative note were marked as 'required' and the op note could only be submitted after all sections have been completed. We also educated senior surgeons on how junior doctors, nurses and other allied professionals, as well as GPs, rely on the accuracy of operation notes to manage patients postoperatively, and therefore the need to avoid the use of abbreviations and provide clear details in the op note. We displayed at our clinical governance meeting the lack of knowledge among junior staff about what certain abbreviations meant. We emphasised that clinical errors arising from the use of abbreviations are well known [15,16], and abbreviations such as ‘R’ (right), ‘L’ (left), NGT (nasogastric tube), EUA (examination under anaesthetic) and LA (local anaesthetic), which were rife in the op notes assessed, may be misinterpreted and could lead to miscommunication and mistakes in patient care [17]. Our intervention led to significant improvement in the use of abbreviations.

The clear advantages of using an electronic proforma are improved legibility, ease of remote access, reduced variability between different operation reports for the same procedure, convenience of tailoring the content to the recommendations of RCS, automatic coding and ease of access for research and audits [10,11]. Disadvantages could be in saving a form due to problem with IT, funding of hardware and software devices and the need for training and familiarisation with the system [5].

## Conclusions

Documenting an accurate and legible operative note is the professional responsibility of every surgeon, as they are vital for post-operative care, remuneration of health care providers and surgeon’s defence in medico-legal inquiries. Trainees should be exposed to formal teaching on the appropriate approach to writing op notes, given that they are responsible for documenting a vast majority of op notes. This audit was able to standardise the operative notes, improve quality and structure, and achieve sustainable change through the implementation of a structured electronic op note proforma.
